# Ice Water Test Unmasks Detrusor Overactivity in Apparently Normal Conventional Cystometry

**DOI:** 10.1016/j.euros.2026.08.004

**Published:** 2026-09-10

**Authors:** Lara Stächele, Helen Sadri, Collene E. Anderson, Veronika Birkhäuser, Oliver Gross, Fabienne Lehner, Lorenz Leitner, Martina D. Liechti, Ulrich Mehnert, Thomas M. Kessler

**Affiliations:** a Department of Neuro-Urology, Balgrist University Hospital, University of Zürich, Zürich, Switzerland; b Spinal Cord Injury Center, Balgrist University Hospital, University of Zürich, Zürich, Switzerland; c Swiss Paraplegic Research, Nottwil, Switzerland; d Faculty of Health Sciences and Medicine, University of Lucerne, Lucerne, Switzerland

**Keywords:** Ice water test, Detrusor overactivity, Urodynamics, Lower urinary tract symptoms, Cystometry

## Abstract

**Background and objective:**

To evaluate the diagnostic value of the ice water test (IWT) in unmasking detrusor overactivity (DO) in patients with lower urinary tract symptoms (LUTS) who do not exhibit DO during conventional filling cystometry and to characterize this specific patient population.

**Design, setting, and participants:**

In this observational cohort study, from April 2021 to December 2023, patients with LUTS but no DO in same-session repeat conventional filling cystometry were additionally evaluated using the IWT. All participants underwent standardized urodynamic investigations following International Continence Society guidelines.

**Outcome measurements and statistical analysis:**

The primary outcome was the presence of DO during the IWT. Demographic and clinical factors associated with DO were analyzed using mixed-effects logistic regression models.

**Results and limitations:**

In 529 UDIs of 451 patients (63 patients had more than one urodynamic investigation (UDI)) without DO in conventional filling cystometry, the IWT revealed DO in 51% (229/451). Detection was more frequent in patients with an underlying neurological disorder (53% vs 34%, *p* = 0.004), males (57% vs 42%, *p* = 0.007), and those using antimuscarinics (67% vs 43%, *p* < 0.001) or being treated with intradetrusor onabotulinumtoxinA injections in the past year (66% vs 46%, *p* = 0.002). These variables, along with increasing age, were independent predictors in a multivariable analysis.

**Conclusions:**

In patients with LUTS but no DO on conventional filling cystometry, the IWT might reveal DO in approximately half of the cases. This underscores the diagnostic value of the IWT in both patients with neurological and nonneurological conditions and leads to a better understanding of LUTS-causing pathophysiology.

## Take Home Message

The ice water test might reveal detrusor overactivity in approximately half of the patients with lower urinary tract symptoms (LUTS) but no detrusor overactivity in conventional filling cystometry, underscoring its diagnostic value in both patients with neurological and nonneurological conditions and contributing to a better understanding of the LUTS causing pathophysiology.

ADVANCING PRACTICE **What does this study add?**

This study demonstrates that the ice water test (IWT) can uncover detrusor overactivity (DO) in approximately half of patients with lower urinary tract symptoms who show no DO during standard filling cystometry. It identifies key clinical predictors of a positive test result, including neurological disease, male sex, and increasing age. By defining clinical characteristics of this subgroup, the findings support the IWT as a complementary diagnostic tool and help refine patient selection for further evaluation and management.

**Clinical Relevance**

The ice water test may complement conventional cystometry when the findings do not explain a patient’s lower urinary tract symptoms, particularly in patients with neurological disease. Demonstration of detrusor overactivity may help clinicians clarify the underlying dysfunction and discuss the findings with patients. However, ice water testing is not standardized, and this study did not determine whether a positive result changes treatment decisions or improves clinical outcomes. Associate Editor: Phe Véronique, MD.

**Patient Summary**

In this study, we tested whether an ice water bladder test can uncover hidden bladder overactivity in patients with urinary symptoms. We found that approximately half of patients who had normal standard test results showed bladder overactivity with this method, especially older patients, men, and those with neurological conditions. These results suggest that this test may help doctors better understand urinary symptoms and choose more targeted treatments.

## 1 Introduction

Urodynamic investigation (UDI) is the only objective measure to evaluate lower urinary tract symptoms (LUTS). Nevertheless, detrusor overactivity (DO) is not always revealed in patients with storage symptoms suggestive of DO [1]. As a urodynamic correlate of storage LUTS, DO is clinically relevant for guiding treatment decisions and predicting therapeutic outcomes [1].

Detailed guidelines exist to ensure high standards in performing UDI regarding measurement techniques, quality, and record-keeping [2]. Furthermore, guidelines have been issued to establish performance benchmarks for urodynamic equipment [3].

Numerous studies have highlighted the importance of the ice water test (IWT) in the diagnosis and characterization of various urological and neurological conditions and thus guiding treatment decisions [4], [5], [6]. Nevertheless, the IWT is not routinely performed by all investigators, and there remains a lack of consensus on its standardized use in clinical practice.

Therefore, we investigated the diagnostic value of the IWT in unmasking DO in patients with LUTS who do not exhibit DO during conventional filling cystometry and characterized this specific patient population.

## 2 Patients and methods

### 2.1 Patients

**Fig. 1:**
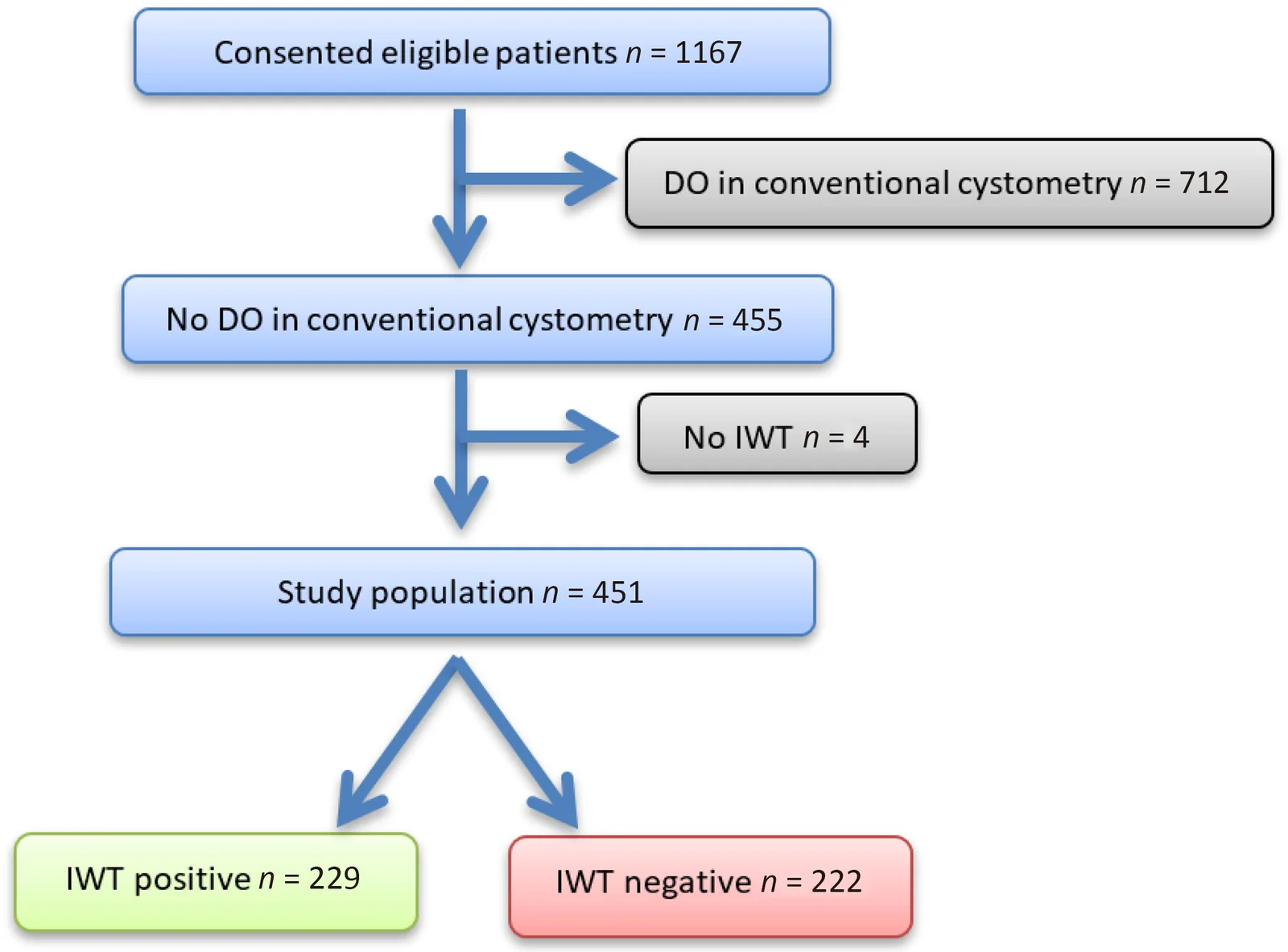
Study population flowchart. DO = detrusor overactivity; IWT = ice water test.

This study was conducted as an observational cohort study of patients undergoing UDI for LUTS at the Department of Neuro-Urology, Balgrist University Hospital, University of Zürich, Zürich, Switzerland, a specialized tertiary referral center, from April 2021 to December 2023. A consecutive series of 1189 patients provided written informed consent; results from the UDI were prospectively collected, and 451 patients who showed no evidence of DO during conventional filling cystometries were included in the study population. Lack of symptoms at the time of UDI was an exclusion criterion. Patients using catheters were considered symptomatic because of the underlying voiding dysfunction and were therefore eligible for inclusion. The study population flowchart is shown in Figure 1. The included patients were aged ≥18 yr. Each patient received a comprehensive neuro-urological assessment, which included a medical history, a clinical examination, and a UDI [7], [8]. The study was approved by the local ethics committee (PB_2016-01089). The methods, definitions, and units used adhere to the standards recommended by the International Continence Society (ICS) [1].

### 2.2 Urodynamics

UDI was conducted in line with Good Urodynamic Practices as advised by the ICS [2]. To assess lower urinary tract function, all patients underwent same-session repeat UDI, including conventional filling cystometry, pressure-flow study for those who could void spontaneously, and pelvic floor electromyography [7], [8]. This was followed by an additional IWT for further evaluation [2], [9]. Whenever feasible, patients underwent urodynamic testing while seated. The filling of the bladder was carried out using saline (0.9% sodium chloride) at 36°C. The repeat UDI within the same session was conducted according to the protocol established in a previous study [10], with modifications regarding the filling rate. Although the original protocol used a filling rate of 20 ml/min, we applied 25 ml/min in this study and adjusted to the bladder capacity measured during the first filling; the filling rates for the second filling were 12 ml/min, 25 ml/min, and 60 ml/min for bladder capacities <350 ml, between 350 and 550 ml, and >550 ml, respectively. During the UDI, blood pressure was continuously monitored [11], [12]. The examination was terminated if there was a strong desire to void, urgency, incontinence, autonomic dysreflexia, pain, or discomfort.

If none of the standard termination criteria applied, filling cystometry was stopped based on individual bladder capacity: in patients without bladder sensation but with a bladder diary, when the filling volume reached the maximum capacity recorded in the bladder diary + the postvoid residual (PVR), if available; in patients without bladder sensation and without a bladder diary, when the filling volume reached 1000 ml; and in patients with bladder sensation, when the volume corresponds to the diary-based capacity + PVR, even if this exceeded 1000 ml.

### 2.3 Study measures

The primary outcome measure was the presence or absence of DO during IWT in cases where DO was not detected during either of the same-session repeat conventional cystometries. In addition, data on demographics (age and sex) and the clinical situation, including diagnosis, medication use, and bladder emptying method, were investigated.

### 2.4 Statistical analyses

In descriptive analyses, continuous variables are presented as medians and first and third quartiles (Q1–Q3), as they were not normally distributed. In the patient characteristic Table 1, differences between LUTS etiology groups (neurogenic vs nonneurogenic) were evaluated using Pearson chi-square tests for sex and categorized age at the first UDI, and univariable logistic (antimuscarinic use, bladder emptying method, storage symptoms, voiding symptoms or catheter use at the time of UDI, and intradetrusor onabotulinumtoxinA injections within 1 yr before UDI) or multinomial logistic (bladder emptying method) regression analyses with robust standard errors allowing for within-patient identification number (ID) clustering. Univariable and multivariable mixed-effects logistic regression analyses were used to generate marginal estimates with 95% confidence intervals (CIs) of the percentage of the population with DO in the IWT and evaluate the associations between DO during the IWT and sex, age (10 yr increase), etiology of LUTS (neurogenic or nonneurogenic), antimuscarinic use at the time of UDI, and intradetrusor onabotulinumtoxinA injections within 1 yr before UDI [7], [8], [13], [14]. Variables were selected a priori for model inclusion based on clinical expert opinion, with support from the literature. A random intercept was placed on the patient ID to account for repeated measures, as some patients underwent multiple UDIs during the study period. The linearity assumption for age was tested using multivariable fractional polynomials. Interactions between all included variables were tested. Missing data in 4% of the patients (16/451) and 3% of the UDIs (17/529) were considered to be missing at random; no imputation was applied. In subgroup analyses of the neurological population, lesion level, categorized as suprasacral, sacral/infrasacral, or mixed, based on diagnosis, replaced LUTS etiology in the regression models. Sensitivity analyses excluding patients with intradetrusor onabotulinumtoxinA injections within 1 yr before UDI and those treated with antimuscarinics at the time of UDI were used to evaluate the robustness of the risk factor identification in the portion of the population that was not already under treatment. Stata version 18.0 (StataCorp, College Station, TX, USA) was used for statistical analyses.

**Table 1:** Characteristics of the study population stratified by etiology of lower urinary tract symptoms

Patients	Neurogenic, *n* (%) 354 (78)	Nonneurogenic, *n* (%) 97 (22)	*p* value^d^
**Sex (female/male)**			0.6
Female	182 (51)	53 (55)	
**Age at first UDI (yr)**			<0.001
18–30	21 (6)	11 (11)	
31–45	64 (18)	33 (34)	
46–60	115 (32)	26 (27)	
61–75	101 (29)	21 (22)	
76+	53 (15)	6 (6)	
**UDIs**	423 (80)	106 (20)	
**Antimuscarinics (at time of UDI; yes/no)**			0.004
Yes	110 (26)	12 (11)	
**OnabotulinumtoxinA up to 1 yr before UDI (yes/no)**			0.4
Yes	70 (17)	13 (12)	
**Bladder emptying method (at time of UDI)**^a^			0.001
Spontaneous	208 (49)	78 (74)	
Combined (spontaneous voiding + catheter)	58 (14)	13 (12)	
Intermittent self-catheterization	106 (25)	10 (9)	
Indwelling catheter	51 (12)	4 (4)	
**Storage symptoms (at time of UDI; yes/no)**^b^			0.7
Yes	190 (45)	50 (47)	
**Voiding symptoms or catheter use (at time of UDI; yes/no)**^c^			0.2
Yes	315 (74)	74 (70)	

UDI = urodynamic investigation.

a Bladder emptying method (*n* = 1).

b Storage symptoms (*n* = 2).

c Voiding symptoms (*n* = 15).

d The *p* values are based on Pearson chi-square tests for “patient-level” characteristics (sex and age at first UDI) and regression analyses with robust standard errors allowing for within-patient clustering in cases with repeated measures.

## 3 Results

During the study period, 451 patients underwent a total of 529 UDIs in which DO was not identified during same-session repeat conventional filling cystometry (Table 1). The median age at first qualifying UDI was 56 yr (Q1–Q3 43–69), and 52% (235/451) of the population was female. Most of the population, 78% (354/451), had neurogenic LUTS. The main neurological disorders were spinal cord injury (*n* = 75), disc herniation/spinal stenosis (*n* = 84), diabetes mellitus (*n* = 26), multiple sclerosis (*n* = 24), and Parkinson disease (*n* = 14).

In total, 14% (63/451) of the patients had more than one UDI included, with 2% (10/451) having three or four qualifying UDIs during the study period. At the time of UDI, 45% of the patients (240/529) had storage symptoms, and/or 74% (389/529) had voiding symptoms (or used a catheter). Patient characteristics and status at the time of UDI stratified by LUTS etiology are presented in Table 1.

Overall, the IWT revealed DO in 51% of the patients (229/451). Of the patients who had repeated visits, 73% (46/63) had consistent IWT results across visits.

Table 2 presents the proportion of patients with DO in the IWT who were stratified only by LUTS etiology, sex, categorized age, antimuscarinic treatment, and within 1 yr after intradetrusor onabotulinumtoxinA injections. DO exclusively during the IWT was more prevalent among patients with neurogenic LUTS than those with nonneurogenic LUTS (53% vs 34%) and in males than in females (57% vs 42%).

**Table 2:** Proportion of the population with DO in the IWT only, stratified by patient characteristics; marginal proportions with 95% CIs and *p* values are obtained from univariable mixed-effects logistic regression, with a random intercept on patient ID to account for repeated measures

Characteristic	Proportion DO in IWT (%; 95% CI)	*p* value
**LUTS etiology**		0.004
Neurogenic	53 (48–58)	
Nonneurogenic	34 (25–44)	
**Sex**		0.007
Female	42 (35–48)	
Male	57 (50–64)	
**Age at UDI (yr)**		0.1
18–30	29 (15–43)	
31–45	45 (35–55)	
46–60	50 (42–59)	
61–75	50 (41–60)	
76+	61 (49–73)	
**Antimuscarinics**		<0.001
No	43 (38–49)	
Yes	67 (58–76)	
**OnabotulinumtoxinA**		0.002
No	46 (41–51)	
Yes	66 (56–76)	

CI = confidence interval; DO = detrusor overactivity; ID = identification number; IWT = ice water test; LUTS = lower urinary tract symptoms; UDI = urodynamic investigation.

In total, 23% of the UDIs involved patients who were using antimuscarinics at the time of assessment (Table 1), and DO only during the IWT had a higher prevalence in these patients compared to those not using antimuscarinics (67% vs 43%; Table 2). Intradetrusor onabotulinumtoxinA injections had been performed in the past year for 16% of the UDIs (Table 1), and these patients had a higher prevalence of DO only during the IWT than assessments that were not done within 1 yr after onabotulinumtoxinA treatment (66% vs 46%; Table 2).

**Fig. 2:**
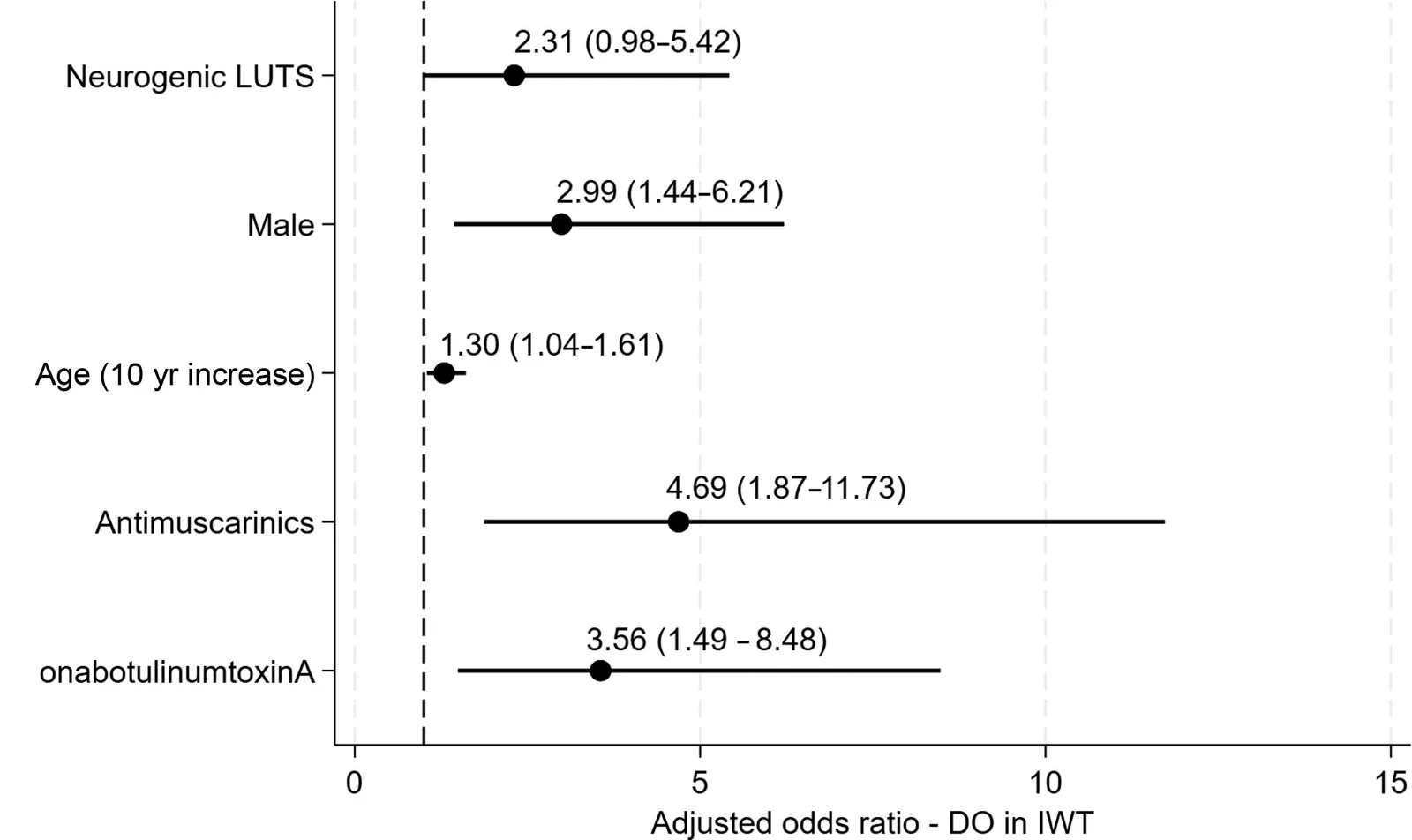
Factors associated with DO during the IWT among patients who did not show DO in conventional filling cystometry. Results from a multivariable mixed-effects logistic regression analysis, with DO during the IWT was coded as “1.” Antimuscarinic usage is the status at the time of UDI; the onabotulinumtoxinA group includes patients with intradetrusor onabotulinumtoxinA injections within 1 yr before UDI. Reference groups for categorical variables: nonneurogenic LUTS (neurogenic LUTS), female (male), no antimuscarinics (antimuscarinics), and no intradetrusor onabotulinumtoxinA (onabotulinumtoxinA). DO = detrusor overactivity; IWT = ice water test; LUTS = lower urinary tract symptoms; UDI = urodynamic investigation.

Multivariable mixed-effects regression analyses confirmed that male sex, neurogenic LUTS, use of antimuscarinics, and intradetrusor onabotulinumtoxinA injections in the past year were all associated with higher odds of having DO during the IWT only (*p* < 0.055 for all; Fig. 2). Age was another factor associated with DO during the IWT: the odds of having DO during the IWT only increased by 30% with every 10-yr increase in age (adjusted odds ratio 1.30, 95% CI 1.04–1.61). In subgroup analyses of the 354 patients with neurogenic LUTS, an estimated 68% (95% CI 61–75%, *n* = 167) of the patients with suprasacral, 43% (95% CI 23–62%, *n* = 27) of those with mixed, and 38% (95% CI 30–45%, *n* = 152) of those with sacral/infrasacral lesions had DO during the IWT (*p* = 0.001). Regression analyses confirmed that patients with diagnoses characterized by suprasacral lesions were more likely to have DO during the IWT than those with sacral/infrasacral lesions (adjusted odds ratio 7.57, 95% CI 2.67–21.46, *p* < 0.0001; Supplementary Table 1). Male sex, older age, and use of antimuscarinics were additional risk factors for DO during the IWT in the neurological population.

Sensitivity analyses limiting the population to a group with a more straightforward interpretation were performed in both the overall population and the neurological subgroup—first excluding patients who had received onabotulinumtoxinA treatment in the past year and then additionally excluding those under antimuscarinic treatment at the time of UDI. Results could be interpreted similarly to the main analysis regarding risk factor identification, however, with more uncertainty regarding the role of neurogenic versus nonneurogenic LUTS in the overall population and increasing age in the most restrictive analysis (Supplementary Tables 2 and 3).

## 4 Discussion

### 4.1 Main findings

As observed in our study, the incorporation of the IWT into routine UDI protocols allowed for the detection of DO in about 50% of the patients, which had not been identified in conventional UDI. This finding aligns with prior research, which emphasizes the sensitivity of the IWT in identifying otherwise undetectable lower urinary tract dysfunction [13], [15], [16]. Thus, our study highlights the importance of integrating the IWT into UDI to uncover DO, particularly in patients with complex urological and neurological conditions, and in those with ambiguous findings, where precise diagnostic insights are essential for tailoring treatment.

### 4.2 Findings in the context of existing evidence

The IWT, first described by Bors and Blinn [20] in 1957, is considered to be based on a C-fiber–mediated reflex triggered by cold stimuli in the bladder wall [17], [18], [19]. The reflex is typically absent in individuals with intact neural pathways but can be unmasked in patients with neurogenic LUTS, serving as a diagnostic indicator of underlying neurological impairment [21]. Several studies [13], [16], [22], [23] have demonstrated that the IWT could unmask DO in patients where conventional UDI failed to do so, making it an indispensable tool in urodynamic protocols. A study by Hentzen et al [24] in patients with multiple sclerosis further demonstrated that urodynamic conditions, including the IWT, may influence bladder sensations and the volume at which DO occurs during cystometry. Differences compared with our findings may be explained by distinct study objectives, the homogeneous multiple sclerosis cohort, and our broader neuro-urological referral population. However, whether DO revealed by the IWT reflects the same underlying pathophysiology as DO during conventional cystometry or represents activation of a distinct reflex pathway without direct symptom relevance remains unclear and requires further investigation. Building on this foundation, our study expands the current knowledge by systematically assessing the value of IWT in both neurogenic and nonneurogenic LUTS, highlighting its diagnostic value in a larger and more diverse patient cohort.

Our cohort included patients who had undergone onabotulinumtoxinA treatment, allowing us to assess the IWT's diagnostic performance across a diverse spectrum of lower urinary tract dysfunctions. The inclusion of this subgroup is particularly important, as previous research has debated whether intradetrusor onabotulinumtoxinA injections alter the results of urodynamic assessments, including the IWT. Our findings suggest that the IWT retains its diagnostic value regardless of prior onabotulinumtoxinA treatment, reinforcing its robustness as a diagnostic tool in the evaluation of DO [23], [25].

Our findings show that male sex and the use of antimuscarinics were independently associated with DO during the IWT. In men, this may be related to structural or functional bladder outlet obstruction, which can promote DO [14]. The association with antimuscarinic use likely reflects more severe or persistent DO. Patients already on treatment may have residual DO that only becomes visible under provocation. Alternatively, it may indicate incomplete suppression of DO by medication.

Moreover, increasing age was independently associated with DO during the IWT, which is consistent with the higher prevalence of DO in older individuals because of age-related changes in the lower urinary tract and the nervous system [26].

### 4.3 Implications for daily practice

The clinical implications of our findings are profound. In routine practice, omitting the IWT would lead to highly relevant underdiagnosis of DO, which means approximately half of our patients would be informed that there is no DO contributing to, and in some patients even fully explaining, their symptoms. This mismatch may puzzle patients, and some will interpret it as “the doctor does not believe me.” Indeed, patients value objective information from UDI and welcome diagnostic insights into the cause of their symptoms [27]. Knowing why symptoms are happening improves patients’ satisfaction and makes patients feel more involved, both crucial requirements for understanding the rationale for treatments [27], [28]. Even if UDI would not change the management, patients reported that knowing what is going on is helpful [27]. However, in most of the cases, UDI findings will be the basis for an optimal treatment strategy. This is particularly critical for refractory neurogenic and nonneurogenic LUTS, as precise diagnostic insights are essential for tailoring interventions. Prior studies, including randomized trials by Kozomara et al [23], have demonstrated that IWT provokes more pronounced detrusor responses compared to standard warm-water filling cystometry. These results, corroborated by our study, support the routine inclusion of IWT in UDI to enhance diagnostic accuracy.

### 4.4 Study limitations

To the best of our knowledge, this study is among the first to explore the occurrence of DO using the IWT in a prospective clinical context; however, it has certain limitations. In contrast to conventional urodynamics, there is no consensus or standardization regarding the IWT, and no normal values are available. As the research was conducted at a specialized neuro-urological center, which tends to treat more severe and complex cases, patients with neurogenic LUTS represented most of the study population, resulting in a comparatively smaller nonneurological subgroup. It is possible that the results of this study will not be transferable to settings with different case mixes. Additionally, the patient group is heterogeneous regarding underlying diagnosis, with the individual diagnostic groups lacking adequate numbers of patients for meaningful incorporation into statistical models or for subgroup analyses.

Severity of LUTS was not systematically assessed using validated questionnaires and could therefore not be correlated with IWT findings. In addition, presence and severity of comorbidities, treatment history (especially treatments received at other clinics), and pretreatment urodynamics (including before antimuscarinic and botulinum toxin treatment) were not consistently reported and could not be reliably included in this analysis. Although UDI is the gold standard to assess refractory LUTS, it remains a nonphysiological investigation, especially when including the IWT; however, no better diagnostic tools are currently available. In this study, we assessed the diagnostic value of the IWT but did not investigate clinical decision-making strategies and treatment management. Thus, the impact of a positive IWT on treatment decisions cannot be determined from these data and would be an important area for future research. In daily clinical practice, we do not interpret IWT findings in isolation but in correlation with symptoms, present and past medical history, comorbidities, and conventional urodynamic outcomes. Together with our patients’ preferences and expectations, these findings form the basis for determining further clinical management.

## 5 Conclusions

Our findings highlight the potential of the IWT in uncovering DO in patients with both neurogenic and nonneurogenic LUTS, in whom conventional filling cystometries appear normal. Especially in patients with neurological conditions, we highly recommend an additional IWT, as it unmasks DO in >50% of the patients. This objective confirmation of DO provides valuable diagnostic information and helps to clarify ambiguous findings from conventional cystometry. However, further prospective studies are needed to evaluate how IWT findings may contribute to a more personalized and evidence-based management of lower urinary tract dysfunction as well as their potential implications for patient care and communication.

  ***Author contributions***: Thomas M. Kessler had full access to all the data in the study and takes responsibility for the integrity of the data and the accuracy of the data analysis.

  *Study concept and design*: Stächele, Sadri, Kessler.

*Acquisition of data*: Stächele, Sadri.

*Analysis and interpretation of data*: Stächele, Sadri, Anderson, Kessler.

*Drafting of the manuscript*: Stächele, Sadri, Kessler.

*Critical revision of the manuscript for important intellectual content*: Anderson, Birkhäuser, Gross, Lehner, Leitner, Liechti, Mehnert.

*Statistical analysis*: Anderson.

*Obtaining funding*: None.

*Administrative, technical, or material support*: None.

*Supervision*: Kessler.

*Other* (specify): None.

  ***Financial disclosures:*** Thomas M. Kessler certifies that all conflicts of interest, including specific financial interests and relationships and affiliations relevant to the subject matter or materials discussed in the manuscript (eg, employment/affiliation, grants or funding, consultancies, honoraria, stock ownership or options, expert testimony, royalties, or patents filed, received, or pending), are the following: None.

  ***Funding/Support and role of the sponsor*:** None.

## Appendix A Supplementary data

Supplementary Data 1 Supplementary tables 1 to 3 (subgroup and sensitivity analyses).
