# Supplementary material for: Ice Water Test Unmasks Detrusor Overactivity in Apparently Normal Conventional Cystometry
Source: Eur Urol Open Sci. 2026 Sep 10;92:64–70. doi: 10.1016/j.euros.2026.08.004 (PMC13584125; doi:10.1016/j.euros.2026.08.004)
Supplement: Supplementary file 1 [file mmc1.docx]

**Table S1. Subgroup analyses: Factors associated with detrusor overactivity (DO) during the ice water test (IWT) among neurological patients who did not show DO in conventional filling cystometry**. Results from uni- and multivariable mixed-effects logistic regression analyses in n=346 neurological patients with DO during the IWT coded as '1'. Medication information reflects status at the urodynamic appointment. Lesion level is categorized based on diagnosis, the suprasacral category includes suprapontine lesions, mixed indicates suprasacral and sacral/infrasacral lesion.

Abbreviations: aOR=adjusted odds ratio; OR=odds ratio; UDI=urodynamic investigation

|  | **Positive IWT,**  **OR (95% CI)** | ***p*** | **Positive IWT,**  **aOR (95% CI)** | ***p*** |
| --- | --- | --- | --- | --- |
| **Lesion level** |  | 0.001 |  | <0.001 |
| Suprasacral | 9.08 (2.77-29.76) |  | 7.57 (2.67-21.46) |  |
| Mixed | 1.46 (0.33-6.41) |  | 1.41 (0.38-5.24) |  |
| Sacral / infrasacral | REF |  | REF |  |
| **Sex** |  | 0.02 |  | 0.005 |
| Female | REF |  | REF |  |
| Male | 2.67 (1.17-6.06) |  | 3.14 (1.40-7.03) |  |
| **Age at UDI [10 years]** | 1.24 (0.98-1.57) | 0.07 | 1.32 (1.04-1.67) | 0.02 |
| **Antimuscarinics** |  | 0.001 |  | 0.002 |
| No | REF |  | REF |  |
| Yes | 4.43 (1.78-11.02) |  | 4.32 (1.70-10.99) |  |
| **OnabotulinumtoxinA** |  | 0.01 |  | 0.1 |
| No | REF |  | REF |  |
| Yes | 3.35 (1.29-8.74) |  | 2.21 (0.88-5.59) |  |

**Table S2. Sensitivity analyses: Factors associated with detrusor overactivity (DO) during the ice water test (IWT) among patients who did not show DO in conventional filling cystometry excluding treated patients**. Results from uni- and multivariable mixed-effects logistic regression analyses with DO during the IWT coded as '1'. Medication information reflects status at the urodynamic appointment. The first sensitivity analysis excluded the patients who were treated with onabotulinumtoxinA up to one year before urodynamic investigation (UDI), n=446 UDI from 398 patients. The second sensitivity analysis excluded the patients on antimuscarinics at the time of urodynamic investigation as well as the onabotulinumtoxinA group, n=344 UDI from 307 patients.

Abbreviations: aOR=adjusted odds ratio; LUTS=lower urinary tract symptoms; OR=odds ratio; UDI=urodynamic investigation

|  | **Positive IWT,**  **OnabotulinumtoxinA**  **Excluded**  **OR (95% CI)** | ***p*** | **Positive IWT,**  **OnabotulinumtoxinA**  **Excluded**  **aOR (95% CI)** | ***p*** | **Positive IWT,**  **OnabotulinumtoxinA**  **& Antimuscarinics**  **Excluded**  **OR (95% CI)** | ***p*** | **Positive IWT,**  **OnabotulinumtoxinA**  **& Antimuscarinics**  **Excluded**  **aOR (95% CI)** | ***p*** |
| --- | --- | --- | --- | --- | --- | --- | --- | --- |
| **LUTS Etiology** |  | 0.006 |  | 0.05 |  | 0.03 |  | 0.07 |
| Non-neurogenic | REF |  | REF |  | REF |  | REF |  |
| Neurogenic | 5.19 (1.62-16.62) |  | 3.20 (1.01-10.11) |  | 3.17 (1.13-8.87) |  | 2.56 (0.91-7.17) |  |
| **Sex** |  | 0.01 |  | 0.006 |  | 0.02 |  | 0.01 |
| Female | REF |  | REF |  | REF |  | REF |  |
| Male | 3.96 (1.34-11.70) |  | 4.69 (1.57-13.96) |  | 3.20 (1.22-8.42) |  | 3.42 (1.28-9.13) |  |
| **Age at UDI [10 years]** | 1.42 (1.06-1.89) | 0.02 | 1.37 (1.01-1.85) | 0.04 | 1.34 (1.01-1.77) | 0.04 | 1.30 (0.98-1.73) | 0.07 |
| **Antimuscarinics** |  | 0.002 |  | 0.003 |  |  |  |  |
| No | REF |  | REF |  | Excluded |  | Excluded |  |
| Yes | 8.88 (2.19-36.02) |  | 8.29 (2.09-32.89) |  | Excluded |  | Excluded |  |

**Table S3. Sensitivity analyses: Neurological subgroup: Factors associated with detrusor overactivity (DO) during the ice water test (IWT) among patients who did not show DO in conventional filling cystometry excluding treated patients**. Results from uni- and multivariable logistic regression analyses with DO during the IWT coded as '1'. Medication information reflects status at the urodynamic appointment. The first sensitivity analysis excluded the patients who were treated with onabotulinumtoxinA up to one year before urodynamic investigation (UDI), n=293 UDI from 293 patients (only 1 observation per patient was included and the random effect was omitted due to convergence issues linked to lack of within-patient variation in the suprasacral group). The second sensitivity analysis excluded the patients on antimuscarinics at the time of urodynamic investigation as well as the onabotulinumtoxinA group, n=254 UDI from 220 patients. Lesion level is categorized based on diagnosis, the suprasacral category includes suprapontine lesions, mixed indicates suprasacral and sacral/infrasacral lesion.

Abbreviations: aOR=adjusted odds ratio; OR=odds ratio; UDI=urodynamic investigation

|  | **Positive IWT,**  **OnabotulinumtoxinA**  **Excluded**  **OR (95% CI)** | ***p*** | **Positive IWT,**  **OnabotulinumtoxinA**  **Excluded**  **aOR (95% CI)** | ***p*** | **Positive IWT,**  **OnabotulinumtoxinA**  **& Antimuscarinics**  **Excluded**  **OR (95% CI)** | ***p*** | **Positive IWT,**  **OnabotulinumtoxinA**  **& Antimuscarinics**  **Excluded**  **aOR (95% CI)** | ***p*** |
| --- | --- | --- | --- | --- | --- | --- | --- | --- |
| **Lesion level** |  | <0.001 |  | <0.001 |  | 0.045 |  | 0.03 |
| Suprasacral | 4.25 (2.55-7.08) |  | 4.55 (2.65-7.80) |  | 11.53 (1.63-81.57) |  | 12.41 (1.95-78.77) |  |
| Mixed | 1.35 (0.56-3.28) |  | 1.38 (0.56-3.39) |  | 0.69 (0.10-4.94) |  | 0.59 (0.08-4.23) |  |
| Sacral / infrasacral | REF |  | REF |  | REF |  | REF |  |
| **Sex** |  | 0.006 |  | 0.001 |  | 0.05 |  | 0.03 |
| Female | REF |  | REF |  | REF |  | REF |  |
| Male | 1.92 (1.21-3.06) |  | 2.36 (1.41-3.93) |  | 2.82 (1.01-7.93) |  | 3.83 (1.15-12.77) |  |
| **Age at UDI [10 years]** | 1.12 (0.97-1.30) | 0.1 | 1.17 (0.99-1.38) | 0.06 | 1.24 (0.90-1.70) | 0.2 | 1.42 (0.98-2.06) | 0.07 |
| **Antimuscarinics** |  | 0.002 |  | 0.005 |  |  |  |  |
| No | REF |  | REF |  | Excluded |  | Excluded |  |
| Yes | 2.35 (1.38-4.01) |  | 2.31 (1.30-4.12) |  | Excluded |  | Excluded |  |
